# Case of Acute Trifacial Neuralgia

**Published:** 1867-07

**Authors:** H. C. Bartleson


### CASE OF ACUTE TRIFACIAL NEURALGIA.

BY H. C. BARTLESON.

Dear Register:—I report the following case because I have never met with another precisely similar.

May 15th—A young lady called who was suffering from an alveolar abscess of considerable size, situate at the roots of the first right superior molar. She was of nervo-sanguine temperament, strumous diathesis, and somewhat anemic. The pain was remittent—during the exacerbations shot along the floor of the orbit, and frequently was frontal and temporal. With some difficulty, owing to their decayed condition, I succeeded in extracting the roots, which was followed by discharges of pus, which led me, notwithstanding the neurotic symptoms, to promise complete relief from pain in a few hours. Only prescribing a topical application of opii vinum.

Four days after she returned with intense intermittent pain in the course of the right superior maxillary and opthalmic division of fifth pair. Augmented heat was apparent in the supra and infra orbital and temporal regions.

The conjunctiva was deeply congested, particularly below the cornea, and epiphora occasioned much annoyance. Mild coryza of that side of nasal passages existed. Symptomatic febrile movement was noticeable.

These symptoms, she said, developed immediately after her last visit, and had been constantly increasing.

But little pain was refered to the situation of the abscess, and the locality had the appearance of a rapid return to the condition of health.

A leach applied to the temple was attended with relief. Tinc. acconiti, chloroform, vinum opii, was directed topically, and pulvis Doveri was prescribed. Doses to be repeated at proper intervals until sleep or an immunity from pain was secured, unless troublesome vomiting intervened.

A cathartic, for existing constipation, entered also into the treatment.

Complete relief from pain was obtained shortly. Refreshing sleep that night was enjoyed the first time for a long period, which was followed by non-continuance of violent paroxysms and rapid improvement.

For the anemia:

Ferri et quinia citras, gr. c.

Syr limonis, ℥ xx.

A teaspoonful to be taken twice and afterwards three times per diem.

The topical application was continued until all pain and soreness disappeared, which was about the sixth day from the time of her second visit. The congestion of the conjunctiva was constantly decreasing for about three weeks. Have seen the patient often since, and there has been no recurrence of any of the symptoms.

There is marked improvement in the general condition. In another case would prefer one of the salts of morphia, for the opiate treatment, to the Dover's powders, although a slight emesis which followed its use was not contra-indicated. I attributel temporary anorexia to its affects.

The ready yielding of the symptoms to such mild measures was gratifying, and but little expected.

In the diagnosis, inflammation of antrum of Highmore, was excluded by the character of the pain, by the absence of continued soreness in that situation, and by the fact that no perforation of that cavity existed.

